#  Comparing Acceptance and Commitment Group Therapy and 12-Steps Narcotics Anonymous in Addict’s Rehabilitation Process: A Randomized Controlled Trial

**Published:** 2016-10

**Authors:** Manoochehr Azkhosh, Ali Farhoudianm, Hemn Saadati, Fateme Shoaee, Leila Lashani

**Affiliations:** 1 University of Social Welfare and Rehabilitation Sciences, Tehran, Iran.; 2 Substance Abuse and Dependence Research Center, University of Social Welfare and Rehabilitation Sciences, Tehran, Iran.; 3 Substance Abuse and Dependence Research Center, University of Social Welfare and Rehabilitation Sciences, Tehran, Iran.; 4 University of Social Welfare and Rehabilitation Sciences, Tehran, Iran.; 5 Student Research Committee, University of Social Welfare and Rehabilitation Sciences, Tehran, Iran.

**Keywords:** *Acceptance and Commitment Therapy*, *Addiction*, *Psychological Flexibility*, *Psychological Well-Being*

## Abstract

**Objective:** Substance abuse is a socio-psychological disorder. The aim of this study was to compare the effectiveness of acceptance and commitment therapy with 12-steps Narcotics Anonymous on psychological well-being of opiate dependent individuals in addiction treatment centers in Shiraz, Iran.

**Method:** This was a randomized controlled trial. Data were collected at entry into the study and at post-test and follow-up visits. The participants were selected from opiate addicted individuals who referred to addiction treatment centers in Shiraz. Sixty individuals were evaluated according to inclusion/ exclusion criteria and were divided into three equal groups randomly (20 participants per group). One group received acceptance and commitment group therapy (Twelve 90-minute sessions) and the other group was provided with the 12-steps Narcotics Anonymous program and the control group received the usual methadone maintenance treatment. During the treatment process, seven participants dropped out. Data were collected using the psychological well-being questionnaire and AAQ questionnaire in the three groups at pre-test, post-test and follow-up visits. Data were analyzed using repeated measure analysis of variance.

**Results:** Repeated measure analysis of variance revealed that the mean difference between the three groups was significant (P<0.05) and that acceptance and commitment therapy group showed improvement relative to the NA and control groups on psychological well-being and psychological flexibility.

**Conclusion**: The results of this study revealed that acceptance and commitment therapy can be helpful in enhancing positive emotions and increasing psychological well-being of addicts who seek treatment.

The number of opiate users in the world is about 1.8-3.3 Million and Iran has the world’s highest proportion of opiate addicts, 2% of the population (15-60 years old (1). The consequences and effects of drug dependency weakens the individual and his/her family socially, economically, physically and psychologically (2). Psychological effects of drug addiction include the lack of willingness, neglect of personal responsibility, character weakness and lack of emotion, nervousness, psychologically imbalance, unbalanced and unstable personality, and low self-esteem (3-5), so addiction reduces psychological well-being (6).

Psychological well-being refers to person’s emotional and cognitive reactions to personal characteristics and skills, effective engagement with the world, appropriate interaction with the community and growth over time (7-9). Six broad facets associated with psychological well-being are self-acceptance, positive relations, environmental mastery, autonomy, purpose in life and sense of personal growth. Self-acceptance means having a positive attitude toward self, awareness and acceptance of various good and bad aspects of self and past life experiences. Positive relationship refers to warm and satisfactory relationship with people, being interested in others’ well-being, having empathy and intimacy to others and understanding of human interaction. Autonomy is self-evaluation based on personal standards and self-control over behaviors. Environmental mastery means control over external events, taking advantages of environmental opportunities and the ability to choose or create a base to meet needs and personal values. Purpose in life is to give meaning to life by setting and achieving goals and in the life (9). 

Living in the moment has a good effect on psychological well-being (10) and mindfulness based interventions is a good way to increase psychological well-being (11). Acceptance and commitment therapy, as a mindfulness intervention, utilizes metaphors and proverbs to help clients improve their life using awareness, acceptance and being in here and now instead of arguments and avoiding internal experiences such as thoughts, memories and feelings (12, 13). 

Acceptance and commitment therapy has two main purposes: Acceptance of problematic internal experiences which cannot be controlled; and commitment and action based on individuals values (14). A part of effectiveness of ACT is under the mindfulness umbrella and present time including acceptance, cognition diffusion, clarifying personal values, action basis on values and commitment in practice (15). 

Also, the twelve-step program is also one of the most widely used methods for quitting drugs. Twelve-steps is a method that uses spiritual orientation based on the confession of personal helplessness and accepting the help of a superior force. It recognizes addiction as a disease and identifies human beings as having three dimensions: Physical, mental and spiritual. Physical symptoms of addiction known as allergy or sensitivity that forces a person to abuse drugs. The psychological effects of addiction are associated with the temptation and spiritual or intellectual abnormalities caused by an over-reliance on self. 

Psychotherapy have a tendency toward positive psychology, and many researchers aim to improve mental health. Considering the emphasis of acceptance and commitment therapy on mindfulness, being in the present moment and acceptance of internal experiences, the aim of this study was to examine the effectiveness of acceptance and commitment therapy on psychological well-being of opiate dependent people in addiction treatment centers in Shiraz. Therefore, this randomized clinical trial (RCT) examined the effect of NA and acceptance and commitment group therapy on psychological well-being of opiate dependents.

## Materials and Method

This was an experimental study with pretest-posttest and follow-up design and control group. The statistical population included opiate dependents in Shiraz who have been admitted to drug rehab centers. Sixty volunteers were selected based on inclusion criteria (age over 18, no psychosis symptoms, informed consent for participation in the study) and assigned to three groups randomly (20 persons per group). One group was randomly selected as the intervention group, one group as NA and another group as a control group. Twelve sessions (every week, 90 minutes per session) of acceptance and commitment group therapy were administered by the corresponding author. Moreover, a course of narcotics anonymous was administered for NA group and the usual methadone treatment was administered for the third group. During the treatment process, seven patients dropped out of the study. Six weeks after the end of therapeutic sessions and post-test, a follow-up was performed. All treatments was free for participants. Two participants were avoided continuing the study (ACT and Methadone groups). 

Psychological welling was measured using the 18-item psychological welling scale. This scale consists of a series of statements reflecting the six areas of psychological well-being: Autonomy, environmental mastery, personal growth, positive relations with others, purpose in life and self-acceptance. Respondents’ responses were rated based on a scale of 1 to 6, with 1 indicating strong disagreement and 6 indicating strong agreement. This scale has good validity and reliability (9). The validity and reliability of this scale was studied in Iran, and the results revealed adequate validity and reliability. Cranach’s alpha for all dimensions of this scale exceeds 0.80 (16). 

Psychological flexibility was measured using AAQ-R. This 19-item scale (7-point Likert scale) was developed by Hayes et al. and it measures acceptance and mindfulness (17); its validity is adequate and its inner reliability is 0.75 through 0.79. The four- month test-retest reliability was 0.64 (18). Internal reliability of AAQ-R in this study is appropriate (Alpha = 0.81).

## Results

The mean age was 27.5 years for ACT group, 28.2 years for NA group and 26.7 years for the control group, respectively. The education mean was 11.2 years for the ACT group, it was 10.3 years for the NA group, 9.8 years for the control group, respectively. Also, history of drug addiction was 5.2 years for the ACT group; it was 4.9 years for the NA group and 5.6 years for the control group, respectively (table 1). 

**Figure1:**
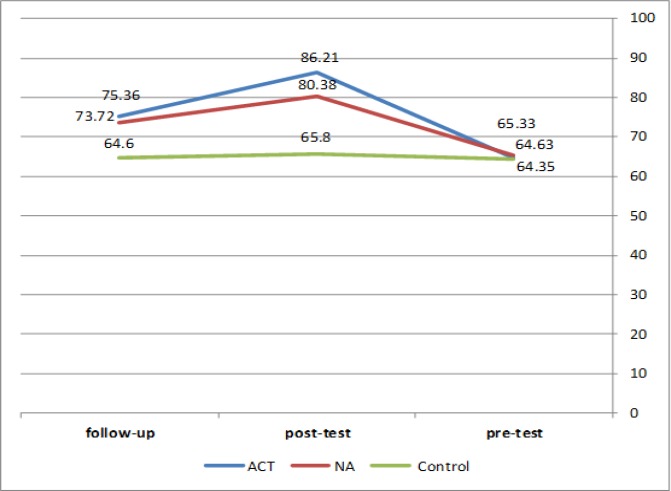
Psychological Well-Being of the Three Measurements in Pre-Test, Post-Test and Follow-Up

The mean of the psychological well-being in the control group had no growth in the three measurements. Psychological well-being of the NA group increased at post-test, but it significantly decreased at follow-up. The psychological well-being of the ACT group increased at post-test and slightly decreased at follow-up (table 2 & figure 1).

**Figure2:**
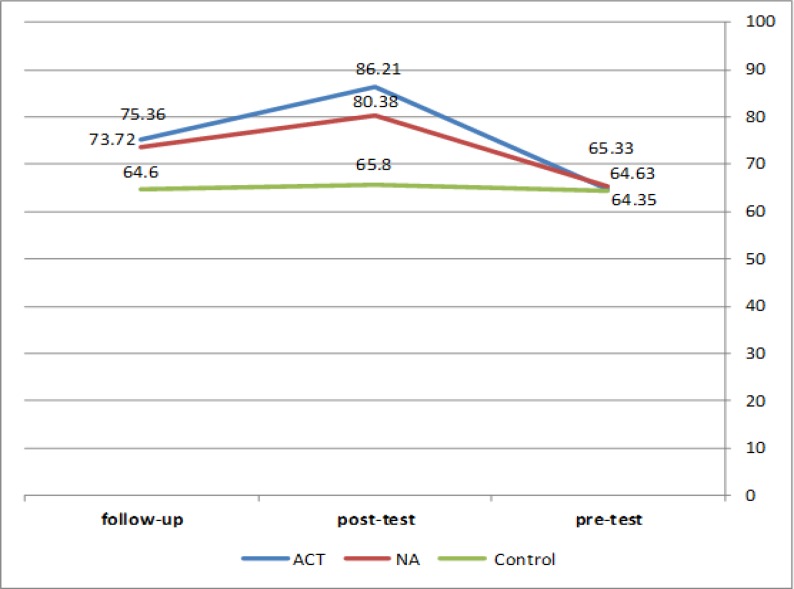
Psychological Flexibility of the Three Measurements in Pre-Test, Post-Test and Follow-Up

No increase was observed in psychological flexibility in the control group at the three measurements. Psychological flexibility increased at post-test and follow-up in ACT group (table 3 & Figure 2). Repeated measure analysis of variance revealed that psychological well-being and psychological flexibility were significantly different between the groups (table 4). Moreover, self-acceptance, purpose in life and personal growth, as psychological well-being subscales, were significantly different between groups (P<0.05).

Bonferoni post-hoc test was used to find out which groups’ mean was significantly different from the others (table 5).

**Table1:** Age, Education and Drug Adiction of the Total Sample

	**ACT(16n )**	**NA(17n)**	**Control(20n)**
The mean age (Sd), y	27.5(7.4)	28.2(6.2)	26.7(7.9)
Education mean (sd), y	11.2(5.4)	10.3(6.1)	9.8(5.9)
History of drug addiction (sd), y	5.2(3.1)	4.9(2.7)	5.6(3.4)

**Table2:** Psychological Well-Being Mean in the Three Measurements of Pre-Test, Post-Test and Follow-Up

**Group**	**Measurement**	**Mean**	**Sd**
ACT N = 16	Pre-test	64.63	**10.06**
	Post-test	86.21	**7.14**
	Follow-up	75.36	**8.02**
NA N = 17	Pre-test	65.33	**6.35**
	Post-test	80.38	**9.17**
	Follow-up	74.94	**7.35**
Control N = 20	Pre-test	64.35	**10.53**
	Post-test	65.80	**10.92**
	Follow-up	64.60	**10.48**

**Table3:** The Mean of Psychological Flexibility of the Three Measurements of Pre-Test, Post-Test and Follow-Up

**Group**	**Measurement**	**Mean**	**Sd**
ACT	Pre-test	67.31	11.03
	Post-test	97.21	14.37
	Follow-up	95.42	18.15
NA	Pre-test	69.27	11.33
	Post-test	81.00	15.71
	Follow-up	78.38	17.17
Control	Pre-test	66.90	11.16
	Post-test	73.15	14.67
	Follow-up	71.45	14.67

**Table4:** Repeated Measures Analysis of Variance to Compare Psychological Well-Being and Psychological Flexibility among the Three Groups in the Three Measurements

**Source**		**SS**	**Df**	**MS**	**F**	**Sig**	**Effect size**
Group	Self-acceptance	245.62	2	122.81	7.10	0.002	0.20
	Positive relations	14.83	2	7.41	0.39	0.67	0.01
	Autonomy	283.45	2	141.72	4.21	0.02	0.13
	Environmental mastery	49.23	2	24.61	1.08	0.34	0.03
	Purpose in life	208.25	2	104.12	12.03	0.001	0.38
	Personal growth	156.53	2	75.26	4.35	0.01	0.13
	Psychological well-being	3111.82	2	1555.91	8.78	0.001	0.24
	Psychological flexibility	6499.03	2	3249.51	9.07	0.001	0.25

*Three Groups: ACT, NA and control group

**Table5:** Bonfferoni Post-Hoc to Compare Groups in Pre-Test, Post-Test and Follow-Up

**Dependent variable**	**Group 1**	**Group 2**	**Mean difference**	**Sig**
Psychological flexibility	Control	ACT	14.81	0.001
		NA	5.72	0.33
Psychological well-being	Control	ACT	9.15	0.001
		NA	8.69	0.003
Self-acceptance	Control	ACT	2.44	0.007
		NA	2.57	0.005
Autonomy	Control	ACT	3.11	0.01
		NA	1.51	0.51
Purpose in life	Control	ACT	2.60	0.001
		NA	0.75	0.51
Personal growth	Control	ACT	0.14	0.9
		NA	2.12	0.03

The results revealed a significant difference in the mean of psychological well-being between the three groups (table 4 & 5). Also, a significant difference was found in psychological flexibility between the control group and the ACT group (table 4 & 5). Both ACT and NA groups were significantly different from the control group in self-acceptance subscale. ACT group was significantly different from the control group in autonomy subscale, but no significant difference was detected between the NA group and the control group in the autonomy subscale. ACT group was significantly different from the control group in purpose of life and personal growth, but no significant difference was found between the NA and control groups in this two subscales.

## Discussion

The hypothesis of this study was that group therapy based on acceptance and commitment therapy can improve psychological well-being and psychological flexibility of addicts in the withdrawal process (table 4 & 5). Our results are similar to those of other studies (19-21) that examined the effect of ACT on outpatient’s mental health and anxiety. 

 ACT is an experienced based psychotherapy that uses awareness and acceptance strategies to change commitment and behavior strategies to achieve psychological flexibility. 

Psychological well-being is reduced when a person is caught regretting the past and having anxiety about the future. Acceptance and commitment therapy helps clients accept their problems and to be aware of their personal values and to behave according to their actual values. All these factors lead to improving self-acceptance, targeting the basis of their values, accepting inner experiences and ultimately improving psychological well-being. 

Acceptance and commitment therapy improves psychological flexibility in addicts in the withdrawal process.

In ACT, the emphasis is on the adoption of internal experiences such as those thoughts and feelings that could end conflict with internal experiences such as cognition or feelings. Initially, accepting what is going through the mind and body can decrease the client’s anxiety (13, 15). 

When an addict encounters with these words from the therapist that it wasn’t useful everywhere you use avoidance and control to be relieved of this inner experiences. Therefore, it is better not to try to run away from these thoughts and feelings. Acceptance is a great stem toward psychological flexibility (22). 

The results revealed that self-acceptance significantly improved in the ACT group, which is similar to the researches that found ACT improves inner world acceptance and self-acceptance (13, 14). Addicts have painful experiences such as punishment, rejection and humiliation that lead to negative thoughts and feelings about themselves. The first wave of cognitive therapy focuses on controlling and avoiding the painful experiences, but ACT recommends the client to experience all bothering experiences and live with them to reach acceptance (14). Also, in group therapy, when members experience mutual respect and acceptance as a human in a group therapy they can respect themselves and improve self-acceptance (23). 

The results showed that autonomy improved in ACT group. A similar study by Weineland et al. (2012) showed that when the person who is receiving acceptance and commitment therapy feels more autonomy and efficacy can enhance emotional or body dissatisfaction (21). Someone who can decide based on his/her thoughts, feelings and personal beliefs is an autonomous person. ACT emphasizes on becoming aware of personal values and act according to inner values (24). Personal values are used as criteria for targeting therapeutic sessions, so ACT can improve purpose in life and targeting according to personal values. Similar studies (25, 26) have found that ACT improve purposing and performance according to personal values.

## Limitations

more studies must be done to confirm efficacy of ACT in treatment of addiction.

## Conclusion

The results of study revealed that both acceptance and commitment therapy and NA improve psychological well-being, but NA cannot improve psychological flexibility. Living in the moment improves psychological flexibility and psychological well-being. Accepting inner experiences improves the clients’ psychological flexibility and their relationship with external experiences. When the client recognizes her/his personal values and acts upon it, he/she can feel more autonomous. 

Considering the sever personal and family problems that substance abusers experience, group therapy and acceptance and commitment therapy could be useful in improving their psychological health and flexibility. Group therapy is a cost effective psychotherapy that can be used for substance abusers and their families. 
